# Isolated Pancreatic Tuberculosis: A Rare Occurrence

**DOI:** 10.4269/ajtmh.2012.12-0054

**Published:** 2012-07-01

**Authors:** Ankur Arora, Amar Mukund, Hitendra Garg

**Affiliations:** Institute of Liver and Biliary Sciences, Radiodiagnosis and Hepatology, New Delhi, India

## Abstract

Isolated tuberculosis of the pancreas is rare even in developing countries where abdominal tuberculosis continues to be prevalent. We present a case of pancreatic tuberculosis in an immunocompetent male with confounding imaging findings and non-contributory clinical details.

A 48-year-old male presented with progressive abdominal discomfort and weight loss of 3 months duration. Laboratory investigations revealed total bilirubin of 3.3 mg/dL, aspartate aminotransferase 141 IU, alanine aminotransferase 100 IU, alkaline phosphatase 436 IU, and erythrocyte sedimentation rate of 78 mm the first hour. Computed tomography revealed a well-marginated cystic lesion in the head of the pancreas with upstream biliary dilatation (Figure 1). The remaining abdominal viscera appeared normal with no abdominal lymphadenopathy. The lesion was subjected to endoscopic ultrasound-guided fine needle aspiration; histology revealed epithelioid granulomas. A polymerase chain reaction (PCR)-based assay confirmed presence of *Mycobacterium tuberculosis* DNA. The patient was initiated on multi-drug anti-tuberculous regimen and remained well 6-months post treatment.

**Figure 1.**
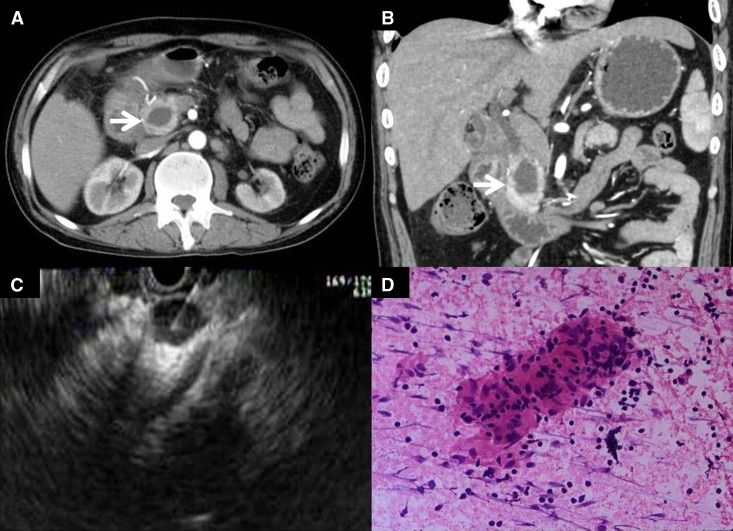
(**A** and **B**) Axial and coronal reconstructed contrast-enhanced computed tomography scan of abdomen showing a hypodense cystic lesion in the head of the pancreas. (**C**) Endoscopic ultrasound-guided fine needle aspiration being performed. (**D**) Hematoxylin and eosin stained histological section (×200) showing epithelioid cell granulomas.

Pancreatic involvement by *M. tuberculosis* is extremely rare, presumably because of the resistance offered by the pancreatic enzymes^1^,^2^; it is thought to be consequential to bacterial dissemination from regional lymph nodes. Imaging manifestations are variable and can mimic pancreatic malignancy or pancreatitis, frequently presenting as a solid or cystic pancreatic mass with or without regional lymphadenopathy.^1^ Findings that suggest a mycobacterial etiology include the presence of centrally necrotic ring-enhancing peripancreatic or mesenteric lymph nodes, presence of ascites, and ileocaecal bowel thickening. Although histopathological illustration of epithelioid cells is highly suggestive, a definitive diagnosis requires demonstration of acid-fast bacilli, isolation of mycobacteria by culture, or molecular detection of mycobacterial DNA in the aspirated material. The majority of the patients respond well to anti-tuberculous therapy.^2^
